# Serious adverse event rates and reoperation after arthroscopic shoulder surgery: population based cohort study

**DOI:** 10.1136/bmj-2021-069901

**Published:** 2022-07-06

**Authors:** Jonathan L Rees, Richard Craig, Navraj Nagra, Mathew Baldwin, Jennifer C E Lane, Andrew Price, David J Beard, Simon Abram, Andrew Judge, Daniel Prieto-Alhambra, Dominic Furniss, Andrew J Carr

**Affiliations:** 1 Nuffield Department of Orthopaedics, Rheumatology and Musculoskeletal Sciences, University of Oxford, Oxford OX3 7LD, UK; 2 NIHR Oxford Biomedical Research Centre, Oxford, UK; 3 Centre for Statistics in Medicine, University of Oxford, Oxford, UK; 4 Musculoskeletal Research Unit, Translational Health Sciences, University of Bristol, Bristol, UK

## Abstract

**Objective:**

To provide clinicians and patients with accurate risk estimates of serious adverse events after common elective shoulder arthroscopic procedures, including reoperation within one year.

**Design:**

Population based cohort study.

**Setting:**

Hospital Episode Statistics for NHS England, including civil registration mortality data from the Office for National Statistics.

**Participants:**

288 250 arthroscopic shoulder procedures performed in 261 248 patients aged ≥16 years between 1 April 2009 and 31 March 2017. Elective procedures were grouped into subacromial decompression, rotator cuff repair, acromioclavicular joint excision, glenohumeral stabilisation, and frozen shoulder release.

**Main outcome measures:**

The primary outcomes were rates of serious adverse events (mortality, pulmonary embolism, pneumonia, myocardial infarction, acute kidney injury, stroke, and urinary tract infection) requiring inpatient care within 90 days post-surgery. Secondary outcomes were specific adverse event rates at 90 days, and reoperations (including for deep infection) within one year.

**Results:**

The overall rate of complications within 90 days after arthroscopic shoulder surgery (including reoperation) was low at 1.2% (95% confidence interval 1.2% to 1.3%), with one in 81 patients at risk, and varied according to type of procedure, from 0.6% (0.5% to 0.8%) for glenohumeral stabilisation to 1.7% (1.5% to 1.8%) for frozen shoulder release. After adjustment for age, comorbidities, and sex, no effect of procedure type was observed. Pneumonia was the most common adverse event (0.3%, 0.3% to 0.4%), with one in 303 patients at risk. Pulmonary embolic events were rare, at 0.1% (0.1% to 0.1%), with one in 1428 patients at risk. At one year, the overall rate for reoperation was 3.8% (3.8% to 3.9%), with one in 26 patients at risk, ranging from 2.7% (2.5% to 3.0%) for glenohumeral stabilisation to 5.7% (5.4% to 6.1%) for frozen shoulder release. The overall rate of further surgery for deep infection was low, at 0.1% (0.1% to 0.1%), with one in 1111 patients at risk, but was higher after rotator cuff repair (0.2%, 0.2% to 0.2%), with one in 526 patients at risk. Over the study period the number of arthroscopic shoulder procedures increased, except for subacromial decompression, which decreased.

**Conclusions:**

The findings of this study suggest that risks of serious adverse events associated with common shoulder arthroscopy procedures are low. Nevertheless, serious complications do occur, and include the risk of reoperation in one in 26 patients within one year.

**Study registration:**

Clinical.Trials.gov NCT03573765.

## Introduction

Because high level evidence in many disciplines often lags behind clinical practice,^1^ it is important for surgeons, doctors, and patients to have accurate estimates of serious adverse events after routine procedures to inform treatment and shared decision making. Elective orthopaedic surgery is now common in most healthcare systems,^2^ driven by musculoskeletal related disability.^3^ The use of arthroscopic (keyhole) surgery has increased rapidly during the past two decades, particularly of the knee^4^ and shoulder.^5^ Minimally invasive surgery such as this is attractive to patients and hospitals because it can be performed without hospital stay (ie, day case), results in small scars and minimal damage to soft tissue, and recovery times are quicker than with standard operations.^6^ Although the complication rate from arthroscopic surgery is considered to be low, published evidence supporting this is limited.^7^ When we conducted a similar analysis of complication rates after shoulder replacement surgery, the rates of adverse events were higher than expected.^8^ Because of the increased frequency of arthroscopic shoulder procedures, a better understanding of the associated complications and adverse events is needed. In some countries, healthcare systems now monitor complication rates and the outcomes of different providers.^9^

The risks of adverse events after the most commonly performed arthroscopic knee intervention, have recently been published,^10^ but no such estimates have been reported for arthroscopic shoulder surgery. This is particularly relevant after the publication of the Can Shoulder Arthroscopy Work? trial^11^ and Finnish Subacromial Impingement Arthroscopy Controlled Trial,^12^ which question the effectiveness of arthroscopic subacromial decompression, another commonly performed procedure. A systematic review found limited data on adverse events after subacromial decompression.^13^ Surgeons, doctors, and patients remain uncertain about the risks associated with subacromial decompression and other arthroscopic shoulder procedures and how these compare with arthroscopic surgery in other joints, such as the knee.^10^ ^14^

Using a comprehensive dataset from Hospital Episode Statistics for England, we estimated the risks of complications within 90 days of the most common elective shoulder arthroscopy procedures, and reoperation within a year. We also compared any increased rates above baseline with those observed after arthroscopic surgery of the knee to determine whether an anatomical location effect exists.^10^

## Methods

### Data source

We used data from the admitted patient care database of Hospital Episode Statistics for NHS England.^15^ Reporting of all inpatient and day case activity to Hospital Episode Statistics is mandatory for NHS funded care and is based on clinical coding by each hospital. Data are stored according to the UK financial year, from 1 April to 31 March. Each hospital stay can comprise one or several finished consultant episodes of treatment. For each episode, procedures are recorded along with dates using the Office for Population Censuses and Surveys Classification of Interventions and Procedures (OPCS-4) codes. Diagnoses for each episode are recorded using the World Health Organization ICD-10 (international classification of diseases, 10th revision) codes. Before release for research, datasets are first pseudonymised by NHS Digital and details are restricted for key demographical and geographical fields. History and follow-up admissions are linkable to index events through pseudonymised identifiers.^16^ The causes and dates of deaths were available from civil registration mortality data provided by the Office for National Statistics.

### Participants

We identified patients aged 16 years or older who underwent an arthroscopic shoulder procedure between 1 April 2009 and 31 March 2017. The start date was chosen to coincide with the introduction of a specific code for subacromial decompression in OPCS-4 (version 4.5). The supplementary file provides full details of the OPCS-4 and ICD-10 codes used to include and exclude patients, to group procedures, and to record comorbidities and complications. We excluded patients with a current diagnosis of primary or secondary malignancy of the shoulder girdle, a history of a shoulder girdle fracture or shoulder operation in the preceding six months, or a history of ipsilateral shoulder replacement surgery at any time. Patients with a glenohumeral dislocation in the preceding six months were excluded, except if the index procedure was stabilisation surgery.

### Data processing

Several OPCS-4 codes can be combined to describe an arthroscopic shoulder procedure. For analysis we grouped procedures according to the dominant treatment procedure code recorded, based on a hierarchy described in the supplementary file. The groups for analysis comprised patients who underwent subacromial decompression, rotator cuff repair, acromioclavicular joint excision, release for frozen shoulder, and stabilisation surgery. The code for subacromial decompression is commonly used with other treatment codes in Hospital Episode Statistics. In this study the subacromial decompression group includes bursectomies but explicitly excludes procedures with additional treatment targets (eg, biceps tenodesis or tenotomy, chondral procedures, synovectomy, acromioclavicular joint excision). We excluded procedures explicitly identified by a revision code.

For each patient identified with an arthroscopic procedure of interest, all previous and subsequent activity for admitted patient care were extracted and associated with the index episode as a linked event or outcome variable. We identified primary diagnoses and systemic adverse events (mortality, pulmonary embolism, pneumonia, myocardial infarction, acute kidney injury, stroke) based on a prespecified list of ICD-10 codes (see supplementary file). Any history of diabetes mellitus was recorded. The Charlson comorbidity index score was calculated as a summary indicator of comorbidity using a validated algorithm.^17^ To be recorded in Hospital Episode Statistics, systemic conditions have to be serious enough to warrant inpatient care.

Hospital Episode Statistics provides additional sociodemographic information on ethnic origin, and on deprivation using the index of multiple deprivation—a measure used in England based on seven domains of deprivation.^18^ Small areas (lower super output areas) are ranked according to the index from least deprived to most deprived. The index of multiple deprivation score recorded for patients in Hospital Episode Statistics are based on place of residence and as such is an indirect measure of individual status.

### Statistical analysis

The primary outcome was rates of serious adverse events (mortality, pulmonary embolism, pneumonia, myocardial infarction, acute kidney injury, stroke, and urinary tract infection) requiring inpatient care within 90 days post-surgery. Secondary outcomes were rates of specific adverse event at 90 days and reoperations (including for infection) within one year.

For participants undergoing procedures on either the right or left shoulders on separate occasions, we presumed that the risk of further surgery was independent. We counted and reported systemic adverse events and reoperations as simple rates (percentages), with corresponding 95% confidence intervals calculated assuming a normal approximation to the Poisson distribution. Event rates were also plotted at 30 day intervals after surgery, up to one year.

The influence of procedure type on adverse outcomes was evaluated by multiple logistic regression adjusted for age, sex, and grouped Charlson comorbidity index score. Continuous variables were preserved. Non-linear relationships were modelled using restricted cubic splines with four default knots placed at the 5th, 35th, 65th, and 95th percentiles. Separate regression models were constructed for the outcomes of any adverse event within 30 days, any reoperation within one year, and reoperation for infection within one year.

Information on age or sex was missing from 0.03% of records. Regardless of the mechanism for missing data, these cases are unlikely to have any meaningful influence on the results and so they were excluded. We also excluded patients with less than 90 days of follow-up and for analyses of reoperation rates at one year we excluded patients with less than one year of follow-up and those with no side recorded for their surgery. Absolute counts of fewer than six individuals are suppressed from reporting in line with NHS Digital guidance.

All data cleaning and pre-processing was performed using Stata MP (Statacorp 2017; Stata Statistical Software: release version 15.0. College Station, TX). All analyses and production of figures was performed using R version 3.4.0 (R Core Team 2017; R Foundation for Statistical Computing, Vienna, Austria).

### Patient and public involvement

In 2015, a James Lind Alliance Priority Setting Partnership on Surgery for Common Shoulder Problems^19^ identified several important questions patients wanted answered. Many of the top 10 questions related to arthroscopic shoulder surgery and so we considered a better understanding of estimated risks with such surgery to be important, particularly as some trials and recommendations have advised against particular types of arthroscopic shoulder surgery.^11^ ^12^ ^20^

## Results

**Fig 1:**
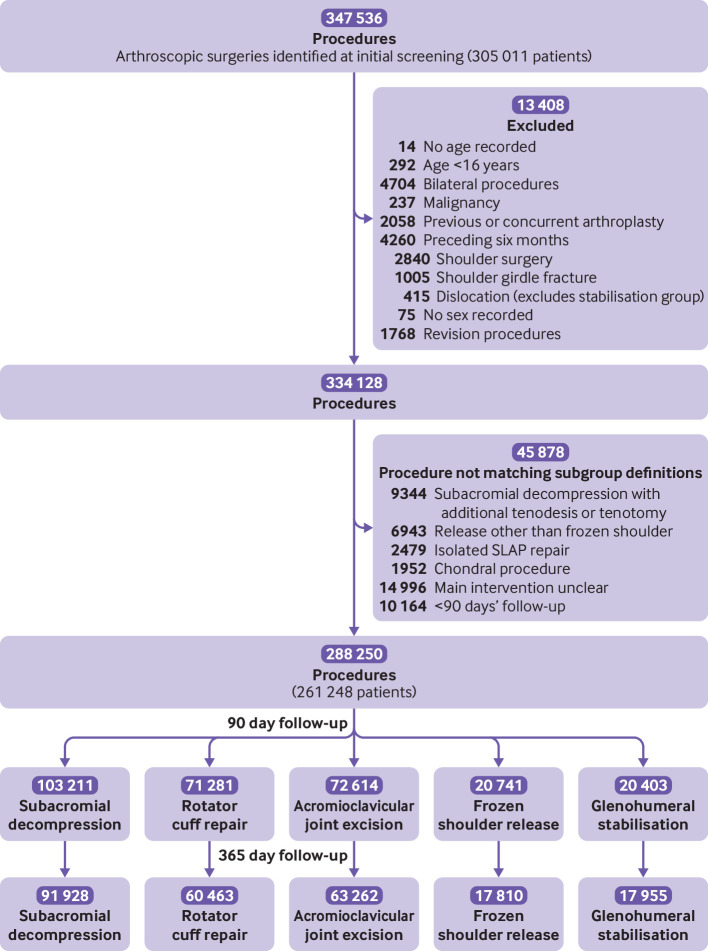
Study flowchart. SLAP=superior labral anterior posterior

**Fig 2:**
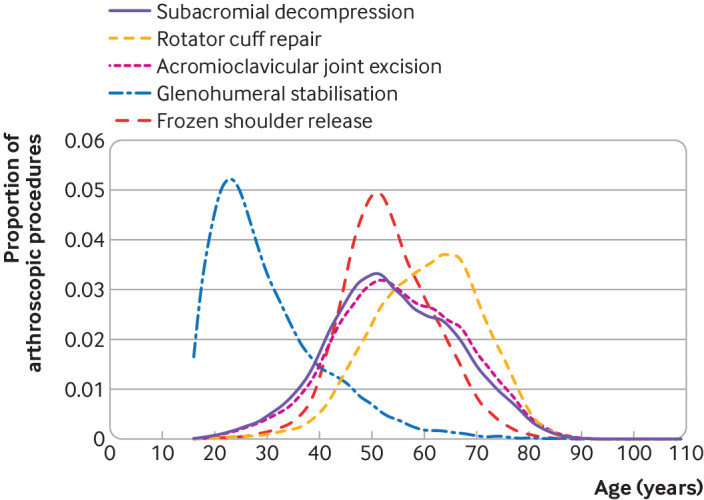
Density plot showing age distributions by type of arthroscopic shoulder procedure

A total of 288 250 arthroscopic shoulder procedures performed in 261 248 patients met the inclusion criteria (fig 1). Over the study period, the number of arthroscopic shoulder procedures increased, except for subacromial decompression, which decreased (see supplementary figure 1). Table 1 shows the characteristics of the patients and figure 2 their age profile by procedure type. The patients undergoing glenohumeral stabilisation surgery were younger, were mostly male (79.4%), and had fewer comorbidities than the patients undergoing the other arthroscopic shoulder procedures. More than one quarter (26.8%) of patients undergoing arthroscopic release for frozen shoulder had diabetes. Two thirds of procedures (66.7%) were performed as day cases.

**Table 1:** Patient characteristics overall and by type of arthroscopic shoulder procedure. Values are numbers (percentages) unless stated otherwise

Characteristics	Overall (n=288 250)	Subacromial decompression (n=103 211)	Rotator cuff repair (n=71 281)	Acromioclavicular joint excision (n=72 614)	Frozen shoulder release (n=20 741)	Glenohumeral stabilisation (n=20 403)
Median (IQR) age (years)	55 (46-64)	53 (46-63)	61 (53-68)	55 (47-64)	53 (47-59)	27 (22-35)
Age (years):						
<25	9086 (3.1)	756 (0.7)	145 (0.2)	427 (0.6)	34 (0.2)	7724 (37.9)
25-34	15 754 (5.5)	4647 (4.5)	713 (1.0)	2826 (3.9)	295 (1.4)	7273 (35.6)
35-44	37 348 (13.0)	17 144 (16.6)	3916 (5.5)	10 485 (14.4)	2590 (12.5)	3213 (15.7)
45-54	80 485 (27.9)	32 470 (31.5)	15 685 (22.0)	21 607 (29.8)	9217 (44.4)	1506 (7.4)
55-64	77 352 (26.8)	26 668 (25.8)	24 346 (34.2)	19 629 (27.0)	6238 (30.1)	471 (2.3)
65-74	52 622 (18.3)	16 316 (15.8)	20 795 (29.2)	13 247 (18.2)	2099 (10.1)	165 (0.8)
75-84	14 776 (5.1)	4905 (4.3)	5449 (7.6)	4120 (5.7)	261 (1.3)	41 (0.2)
≥85	827 (0.3)	305 (0.3)	232 (0.3)	273 (0.4)	7 (0.1)	10 (0.1)
Sex:						
Male	150 451 (52.2)	49 586 (48.0)	40 087 (56.2)	35 801 (49.3)	8767 (42.3)	16 210 (79.4)
Female	137 799 (47.8)	53 625 (52.0)	31 194 (43.8)	36 813 (50.7)	11 974 (57.7)	4193 (20.6)
Ethnicity:						
White	236 860 (82.2)	85 772 (83.1)	57 143 (80.2)	61 164 (84.2)	17 158 (82.7)	15 623 (76.6)
Asian	9867 (3.4)	3178 (3.1)	3120 (4.4)	1989 (2.7)	676 (3.3)	904 (4.4)
Black	4823 (1.7)	1535 (1.5)	1590 (2.2)	1015 (1.4)	167 (0.8)	516 (2.5)
Mixed	1675 (0.6)	513 (0.5)	403 (0.6)	335 (0.5)	84 (0.4)	340 (1.7)
Other	3037 (1.1)	1071 (1.0)	787 (1.1)	573 (0.8)	186 (0.9)	420 (2.1)
Unknown	31 988 (11.1)	11 142 (10.8)	8238 (11.5)	7538 (10.4)	2470 (11.9)	2600 (12.7)
Charlson comorbidity index score:						
0	187 694 (65.1)	68 778 (66.6)	44 736 (62.8)	45 761 (63.0)	11 574 (55.8)	16 845 (82.6)
1	65 779 (22.8)	22 837 (22.1)	16 689 (23.4)	17 371 (23.9)	5734 (27.6)	3148 (15.4)
≥2	34 777 (12.1)	11 596 (11.3)	9856 (13.8)	9482 (13.1)	3433 (16.6)	410 (2.0)
Diabetes	33 872 (11.8)	10 274 (10.0)	8267 (11.6)	8191 (11.3)	5562 (26.8)	334 (1.6)
Index of multiple deprivation fifth:						
1 (least deprived)	59 112 (20.5)	20 734 (20.1)	14 621 (20.5)	15 551 (21.4)	4282 (20.6)	3924 (19.2)
2	61 031 (21.2)	22 033 (21.3)	15 462 (21.7)	15 486 (21.3)	4191 (20.2)	3859 (18.9)
3	56 205 (19.5)	20 407 (19.8)	13 753 (19.3)	13 949 (19.2)	4003 (19.3)	4093 (20.1)
4	54 828 (19.0)	19 406 (18.8)	13 557 (19.0)	13 801 (19.1)	3999 (19.3)	4065 (19.9)
5 (most deprived)	54 322 (18.8)	19 801 (19.2)	13 242 (18.6)	13 082 (18.0)	4097 (19.8)	4100 (20.1)
Not recorded	2752 (1.0)	830 (0.8)	646 (0.9)	745 (1.0)	169 (0.8)	362 (1.8)
Length of hospital stay:						
Day case	192 278 (66.7)	71 917 (69.7)	40 989 (57.5)	51 597 (71.1)	13 974 (67.4)	13 801 (67.6)
1 night	82 010 (28.4)	26 861 (26.0)	25 735 (36.1)	18 005 (24.8)	5540 (26.7)	5869 (28.8)
2 nights	7365 (2.6)	2320 (2.2)	2463 (3.5)	1471 (2.0)	727 (3.5)	384 (1.9)
≥3 nights	6597 (2.3)	2113 (2.1)	2094 (2.9)	1541 (2.1)	500 (2.4)	349 (1.7)

IQR=interquartile range.

Table 2 presents the unadjusted rates of adverse events within 90 days post-surgery by type of arthroscopic shoulder procedure. Table 3 presents the rates of specific adverse events within 90 days post-surgery. The overall risk of adverse events requiring inpatient care or reoperation within the first 90 days was 12.0 per 1000 procedures (95% confidence interval 11.9 to 12.7 per 1000). The likelihood of an adverse event varied according to type of arthroscopic procedure, from 6 per 1000 patient procedures (5.4 to 7.6 per 1000) for stabilisation surgery up to 17 per 1000 patient procedures (14.8 to 18.3 per 1000) for frozen shoulder release. After adjusting for age, sex, and comorbidities, however, no clear differences were observed between the groups (model presented in supplementary materials).

**Table 2:** Unadjusted rates of adverse events within 90 days after arthroscopic surgery procedures

Adverse events	Overall (n=288 250)	No of patients (%, 95% CI)				
		Subacromial decompression (n=103 211)	Rotator cuff repair (n=71 281)	Acromioclavicular joint excision (n=72 614)	Frozen shoulder release (n=20 741)	Glenohumeral stabilisation (n=20 403)
Death	151 (0.05, 0.04 to 0.06)	61 (0.06, 0.05 to 0.08)	32 (0.04, 0.03 to 0.06)	45 (0.06, 0.04 to 0.08)	7 (0.03, 0.01 to 0.06)	6 (0.03, 0.01 to 0.07)
Reoperation	1275 (0.44, 0.42 to 0.46)	418 (0.40, 0.36 to 0.44)	304 (0.43, 0.38 to 0.48)	286 (0.39, 0.35 to 0.44)	195 (0.94, 0.82 to 1.08)	72 (0.35, 0.28 to 0.44)
Systemic adverse event	2297 (0.80, 0.77 to 0.83)	773 (0.75, 0.70 to 0.80)	668 (0.94, 0.87 to 1.01)	649 (0.89, 0.82 to 0.96)	148 (0.71, 0.60 to 0.83)	59 (0.29, 0.22 to 0.37)
Systemic event or reoperation	3546 (1.23, 1.19 to 1.27)	1186 (1.15, 1.09 to 1.22)	959 (1.35, 1.27 to 1.44)	929 (1.28, 1.20 to 1.37)	342 (1.65, 1.48 to 1.83)	130 (0.64, 0.54 to 0.76)

CI=confidence interval.

**Table 3:** Rates of specific adverse events within 90 days after arthroscopic shoulder procedures

Adverse events	Overall (n=288 250)		No of patients (%, 95% CI)			
		Subacromial decompression (n=103 211)	Rotator cuff repair (n=71 281)	Acromioclavicular joint excision (n=72 614)	Frozen shoulder release (n=20 741)	Glenohumeral stabilisation (n=20 403)
Death	151 (0.05, 0.04 to 0.06)	61 (0.06, 0.05 to 0.08)	32 (0.04, 0.03 to 0.06)	45 (0.06, 0.04 to 0.08)	7 (0.03, 0.01 to 0.06)	6 (0.03, 0.01 to 0.07)
Pulmonary embolism	199 (0.07, 0.06 to 0.08)	63 (0.06, 0.05 to 0.08)	73 (0.10, 0.08 to 0.13)	49 (0.07, 0.05 to 0.09)	10 (0.05, 0.03 to 0.09)	4 (0.02, 0.01 to 0.05)
Myocardial infarction	323 (0.11, 0.10 to 0.12)	112 (0.11, 0.09 to 0.13)	97 (0.14, 0.11 to 0.17)	93 (0.13, 0.11 to 0.16)	19 (0.09, 0.06 to 0.14)	2 (0.01, 0.00 to 0.04)
Pneumonia	938 (0.33, 0.31 to 0.35)	296 (0.29, 0.26 to 0.32)	280 (0.39, 0.35 to 0.44)	280 (0.39, 0.35 to 0.44)	56 (0.27, 0.21 to 0.35)	26 (0.13, 0.09 to 0.19)
Urinary tract infection	544 (0.19, 0.17 to 0.21)	188 (0.18, 0.16 to 0.21)	149 (0.21, 0.18 to 0.25)	156 (0.21, 0.18 to 0.25)	34 (0.16, 0.11 to 0.22)	17 (0.08, 0.05 to 0.13)
Acute kidney injury	274 (0.10, 0.09 to 0.11)	79 (0.08, 0.06 to 0.10)	90 (0.13, 0.11 to 0.16)	67 (0.09, 0.07 to 0.11)	30 (0.14, 0.10 to 0.20)	8 (0.04, 0.02 to 0.08)
Cerebrovascular event	212 (0.07, 0.06 to 0.08)	72 (0.07, 0.06 to 0.09)	63 (0.09, 0.07 to 0.12)	61 (0.08, 0.06 to 0.10)	12 (0.06, 0.03 to 0.11)	4 (0.02, 0.01 to 0.05)
Reoperation	1275 (0.44, 0.42 to 0.46)	418 (0.40, 0.36 to 0.44)	304 (0.43, 0.38 to 0.48)	286 (0.39, 0.35 to 0.44)	195 (0.94, 0.82 to 1.08)	72 (0.35, 0.28 to 0.44)
Systemic adverse event	2297 (0.80, 0.77 to 0.83)	773 (0.75, 0.70 to 0.80)	668 (0.94, 0.87 to 1.01)	649 (0.89, 0.82 to 0.96)	148 (0.71, 0.60 to 0.83)	59 (0.29, 0.22 to 0.37)
Systemic event or reoperation	3546 (1.23, 1.19 to 1.27)	1186 (1.15, 1.09 to 1.22)	959 (1.35, 1.27 to 1.44)	929 (1.28, 1.20 to 1.37)	342 (1.65, 1.48 to 1.83)	130 (0.64, 0.54 to 0.76)

CI=confidence interval.

**Fig 3:**
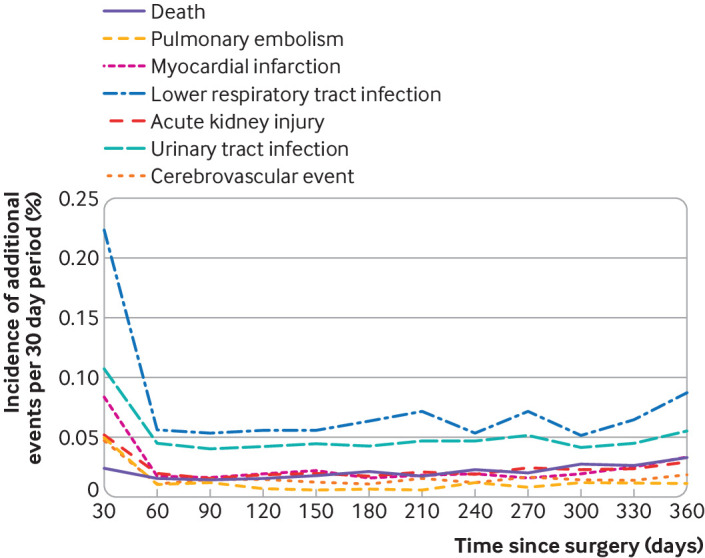
Incidence of additional adverse events within first year post-surgery

The incidence of death within 90 days was low (5 per 10 000 patient procedures, 95% confidence interval 4 to 6 per 10 000). The most commonly recorded adverse event was pneumonia (3 per 1000 patient procedures, 3.1 to 3.5 per 1000). Table 4 shows the results for specific adverse events. When plotting the temporal relationship between adverse events and arthroscopic surgery over the first year, no association was observed between death and surgery (fig 3). Each of the systemic events of interest were most frequent in the first 30 days after surgery and then decreased to a stable baseline rate within 90 days. The relative risks of a complication occurring in the first 30 days compared with any 30 day interval between day 90 and one year were: 5.7 (95% confidence interval 4.5 to 7.1) for pulmonary embolism, 4.0 (3.4 to 4.7) for myocardial infarction, 3.5 (3.2 to 3.9) for pneumonia, 2.4 (2.1 to 2.7) for urinary tract infection, 2.4 (2.0 to 2.9) acute kidney disease, and 3.3 (2.7 to 4.1) for stroke.

**Table 4:** Risk of reoperation within one year after arthroscopic shoulder procedures

Reoperation type	Overall (n=258 363)	No of patients (%, 95% CI)				
		Subacromial decompression (n=94 819)	Rotator cuff repair (n=62 291)	Acromioclavicular joint excision (n=64 873)	Frozen shoulder release (n=18 184)	Glenohumeral stabilisation (n=18 196)
Any reoperation	9877 (3.82, 3.75 to 3.90)	3855 (4.07, 3.94 to 4.20)	1898 (3.05, 2.92 to 3.19)	2588 (3.99, 3.84 to 4.15)	1039 (5.71, 5.37 to 6.07)	497 (2.73, 2.50 to 2.98)
Reoperation for deep infection	225 (0.09, 0.08 to 0.10)	51 (0.05, 0.04 to 0.07)	120 (0.19, 0.16 to 0.23)	40 (0.06, 0.04 to 0.08)	<6 (<0.03%)	<12 (<0.07%)
Arthroplasty	983 (0.38, 0.36 to 0.40)	398 (0.42, 0.38 to 0.46)	188 (0.30, 0.26 to 0.35)	324 (0.50, 0.45 to 0.56)	64 (0.35, 0.27 to 0.45)	9 (0.05, 0.03 to 0.10)

CI=confidence interval.

The likelihood of reoperation at one year after arthroscopic shoulder surgery was 3.8% overall (95% confidence interval 3.8% to 3.9%). Further surgery for deep infection was rare (9 per 10 000 patient procedures, 95% confidence interval 8 to 10), and arthroplasty was performed in four of 1000 (95% confidence interval 3.6 to 4.6) arthroscopic shoulder surgeries (excluding glenohumeral stabilisation). Table 4 reports rates by procedure type. Further surgery was less common after glenohumeral stabilisation and more common after frozen shoulder release (5.7%, 95% confidence interval 5.4% to 6.1%). The effect of age, sex, and comorbidities on the likelihood of any further surgery was examined in a regression analysis stratified by procedure type. No clear associations beyond the effect of procedure type were identified. A wide range of further arthroscopic and open surgeries was performed, but some patterns emerged that were specific to the type of procedure. After rotator cuff repair, 1.3% of patients underwent a further rotator cuff repair operation within one year. For patients undergoing a frozen shoulder release, 3.6% required a further frozen shoulder release or manipulation under anaesthesia, and after an acromioclavicular joint excision 1.3% underwent a repeat excision.

Surgery for deep infection within one year was associated with male sex (odds ratio 5.26, 95% confidence interval 3.6 to 7.7), rotator cuff repair (2.7, 2.0 to 3.8, compared with subacromial decompression), and increasing age (2.1, 1.4 to 3.2, for contrast of 46 versus 64 years on a continuous non-linear scale) (see supplementary file). However, even for a patient with all adverse characteristics (male patient older than 64 years undergoing a rotator cuff repair) the absolute infection rate was still low (<5 in 1000 patient procedures).

## Discussion

This study found a substantial temporal increase in some elective arthroscopic shoulder procedures, and a decrease in the previously most common procedure of subacromial decompression. Overall, the rate of adverse events (excluding reoperation) after arthroscopic shoulder surgery in patients aged 16 years and older is low (one in 81), and although the overall likelihood of an event varied according to type of procedure, these differences seem to be accounted for by age, comorbidity profile, and sex of the patients. In contrast with a low overall risk of serious adverse event, reoperation was more frequent. One in 26 patients underwent reoperation within one year, suggesting either an ineffective procedure or a complication. The reoperation rate varied by procedure type, from one in 18 patients for frozen shoulder release to one in 37 people for glenohumeral stabilisation. Particularly high reoperation rates were observed after frozen shoulder release and this probably highlights the poorly understood and unpredictable nature of this condition.

### Strengths and weaknesses of this study

A strength of this study is the large sample size. Using large volume population level data ensures more precise estimates of incident rates of adverse events and reoperation rates that are representative of real world national outcomes. This study benefits from universal coverage of a national healthcare system and is at less risk of confounding from local geographical, socioeconomic, and commissioning factors than studies from single units or regions.

Although no standard reference dataset for analysing adverse events exists, those reported in this study were severe enough to warrant hospital admission and inpatient care. Our study therefore did not capture complications that were mild enough to be treated in primary care. Similarly, the estimates we present for venous thromboembolism are an underestimate of all events as they only represent the rate of those needing hospital admission and not the many instances of deep vein thromboses that would have been treated without hospital admission and not recorded. We also found an increased rate of pneumonia after arthroscopic shoulder surgery. Although Hospital Episode Statistics data only represent those treated in hospital and the actual overall rate of pneumonia could be higher, the inpatient cases we have captured are likely to be more serious, which further highlights the importance of this finding and the need to discuss this complication with patients before surgery.

### Comparison with other studies

The number of many arthroscopic shoulder procedures are increasing, with patients opting for these types of operation despite limited evidence of effectiveness in some cases,^12^ ^21^ and a lack of reliable data on serious adverse events and reoperation rates.^22^ ^23^ Our findings are therefore overdue and important and will better inform patients, clinicians, and healthcare providers.

The 90 day risks associated with the most commonly performed arthroscopic knee procedure were recently published using the same methods as this study.^10^ Recent trials 12 21 on subacromial decompression shoulder surgery have led to new rapid recommendations^20^ and a reduction in the use of this procedure,^24^ but as the numbers of complications from arthroscopic interventions in trials tend to be small, obtaining reliable rates of adverse events for subacromial decompression and other arthroscopic shoulder procedures has been difficult. This means that attempts to provide estimates for serious adverse events after shoulder arthroscopy have been through systematic reviews.^22^ ^23^ Trial data enabling an assessment of harms for rotator cuff repair have been insufficient, and two observational studies provided an estimate of serious adverse events after subacromial decompression surgery.^13^ Unlike with our study, none of these studies provided complications by procedure type.^25^ ^26^ No additional studies were identified in our updated search (10 September 2021) following the published search strategies for the previous meta-analyses of harms.

In orthopaedic surgery, prevention of venous thromboembolism is important, especially in patients undergoing spinal and lower limb surgery, where venous thromboembolism has always been considered more common. This study confirms a low rate of pulmonary embolism requiring hospital admission (one in 1428 patients) for upper limb surgery, and the risk observed is the same as that seen after arthroscopic partial meniscectomy of the knee.^10^ An unexpected finding was the high rate of pneumonia, observed in one in 303 patients in our study. This rate was higher with increasing age and higher than that seen after arthroscopic partial meniscectomy procedures.^10^ The reasons for this are unknown, but possibilities include shoulder pain (before or after surgery), use of a sling, or anaesthetic methods utilising nerve blocks that can transiently affect the phrenic nerve, all of which might affect deep ventilation and increase the risk of pneumonia in some patients.

In our study, we found that reoperation for deep infection was rare (one in 1111 patients). This rate was less than that observed in a knee arthroscopy cohort^10^ but did vary by procedure type, age, and sex, with the highest risk among male patients undergoing rotator cuff repair. Small implant anchors are used for rotator cuff repair, but despite similar implants being used in shoulder stabilisation procedures, the rate of deep infection was not higher in this last group. Patients who require shoulder stabilisation procedures are, however, younger, suggesting that age (and therefore fewer comorbidities) and not just the use of an implant could play a part in the outcome.

### Meaning of the study

Although the temporal trends observed showed a decrease in subacromial decompression surgeries, the rates of the remaining arthroscopic shoulder procedures continue to increase. Until further high quality evidence on the effectiveness of other procedures is available, we have presented the risk of serious adverse events that require hospital readmission after the most common elective shoulder arthroscopy operations. Overall, the rates were observed to be low (but not absent), although reoperation within one year is relatively high (one in 26 patients). In contrast with established surgical beliefs, some serious adverse events such as pulmonary embolism can occur after routine shoulder arthroscopy. We also found that the rates of adverse events after arthroscopic surgery seem to be joint specific and vary between types of procedure and between the patient groups presenting for different procedures. We found that the infection rate in shoulder arthroscopy was higher for those undergoing rotator cuff repair compared with other arthroscopic shoulder procedures. Although the overall rate of pulmonary embolism after arthroscopic shoulder procedures was the same as that observed after arthroscopic partial meniscectomy of the knee,^10^ the reoperation rate for deep infection was 50% less, whereas the pneumonia rate was 300% higher. As the numbers of other arthroscopic shoulder procedures continue to increase, this study provides real world generalisable estimates of serious adverse events and reoperation rates that should better inform surgeons and patients.

### Unanswered questions and future research

The reasons for the observation of an increased rate of pneumonia within 90 days of arthroscopic shoulder surgery compared with arthroscopic knee surgery are unclear and therefore further research would be needed to identify causation and preventive measures. Further research should consider factors associated with the increased infection rates after rotator cuff repair, along with preventive measures.

**What is already known on this topic**

Arthroscopic shoulder surgery is becoming increasingly common, yet evidence from randomised controlled trials and adverse event data are lacking
As the numbers of complications from arthroscopic interventions in trials tend to be small, obtaining reliable rates of adverse events is difficult and has been through systematic review methods
Cochrane reviews of subacromial decompression and rotator cuff repair were published in 2019 but trial data were insufficient to provide an assessment of associated adverse events

**What this study adds**

The findings of this study suggest a low overall rate of adverse events within 90 days after arthroscopic shoulder surgery (including reoperation), with one in 81 patients at risk
The most common adverse event was pneumonia (one in 303), and pulmonary embolism was rare (one in 1428 patients)
One in 26 patients required reoperation at one year, with a low rate for infection (one in 1111 patients) but higher for rotator cuff repair (one in 526)

## Data Availability

The study is based on NHS Hospital Episode Statistics data and was provided within the terms of an NHS Digital data sharing agreement. The data do not belong to the authors and may not be shared by the authors, except in aggregate form for publication. Data can be obtained by submitting a research request via the NHS Digital Data Access Request Service.

## Web extra

Extra material supplied by authors

## Notes

Contributors: JLR is the first and corresponding author and responsible for the concept, study design, data interpretation, and original and final drafts. RC and JCEL contributed to study design, data analysis, data interpretation, and the original draft. NN and MB contributed to the literature search and original draft. AP, DJB, SA, and AJ contributed to study design, data interpretation, review, and editing. DPA and DF contributed to data interpretation, review, and editing. AJC contributed to data interpretation and the original draft. All authors have read and approved the final manuscript. JLR is the guarantor. The corresponding author attests that all listed authors meet authorship criteria and that no others meeting the criteria have been omitted.

The manuscript’s guarantor (JLR) affirms that this manuscript is an honest, accurate, and transparent account of the study being reported; that no important aspects of the study have been omitted; and that any discrepancies from the study as planned have been explained.

Dissemination to participants and related patient and public communities: The results of the study will be disseminated to stakeholders through national orthopaedic conferences, journals, and websites. We will disseminate the findings to wider audiences and the public through other platforms such as social media.

Provenance and peer review: Not commissioned; externally peer reviewed.
