# Supplementary material for: Serious adverse event rates and reoperation after arthroscopic shoulder surgery: population based cohort study
Source: BMJ. 2022 Jul 6;378:e069901. doi: 10.1136/bmj-2021-069901 (PMC9258605; doi:10.1136/bmj-2021-069901)
Supplement: Supplementary file 1 — Supplementary information: additional tables 1-3 and figures 1-4 [file reej069901.ww.pdf]

Supplementary materials

Supplementary figure 1: Number of arthroscopic shoulder procedures performed per financial year 2010 to 2017

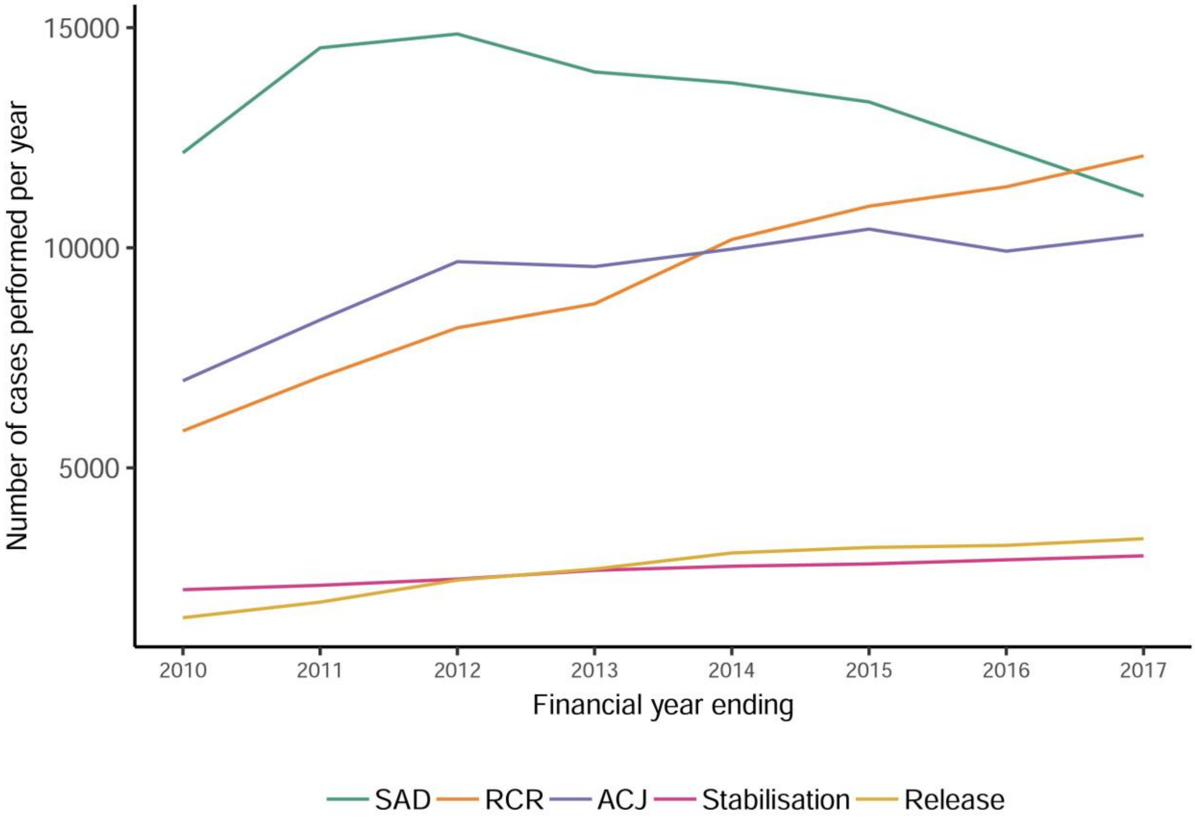

Risk of any adverse events within 30 days – regression

Outcome: Any adverse event including reoperation
Variables: Age, procedure type, sex, grouped Charlson index
Sample size: 288 250
Events: 1782
Degrees of freedom in final model: 10

Age was modelled with splines. Knots at 28, 50, 60 and 75 years. Non-linear effects of age were significant and preserved. There was no significant interaction between age and procedure type. Inclusion of ethnic origin and IMD did not significantly alter the coefficients and did not improve the models (Likelihood ratio test Chi-squared 10.047 on 6 degrees of freedom, p=0.123).

Supplementary table 1: Coefficients from regression for any adverse event within 30 days

Variable	Odds ratio (95% CI)
Age (46 vs 64 years)	1.38 (1.20 to 1.58)
SAD	Reference
RCR	1.09 (0.97 to 1.23)
ACJ	1.07 (0.95 to 1.21)
Stabilisation	1.17 (0.85 to 1.63)
Release	0.90 (0.73 to 1.10)
Male	Reference
Female	0.73 (0.66 to 0.80)
Charlson 0	Reference
Charlson 1	1.59 (1.42 to 1.78)
Charlson 2	2.74 (2.44 to 3.08)

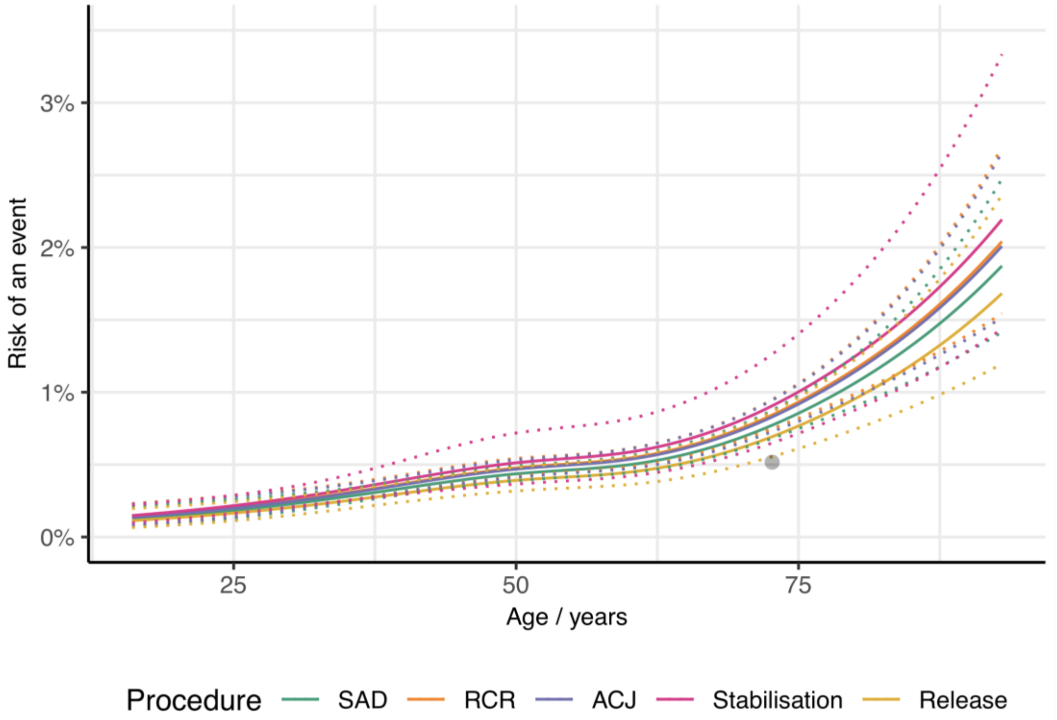

Supplementary figure 2: Non-linear effect of age on risk of adverse events within 30 days, adjusted to male sex and Charlson Index of 0.

Risk of reoperation within 365 days – regression

Outcome: Any reoperation on ipsilateral shoulder within 365 days
Variables: Age, procedure type, sex, grouped Charlson index
Sample size: 251 418
Events: 10 091
Degrees of freedom in final models: 6

Age was modelled with splines. Non-linear effects of age were significant and preserved. When an initial model was fit, there was a significant interaction between age and procedure type. Therefore, five separate models were fit for each of the procedure types rather than reporting a model with interaction terms since the interpretation is more intuitive.

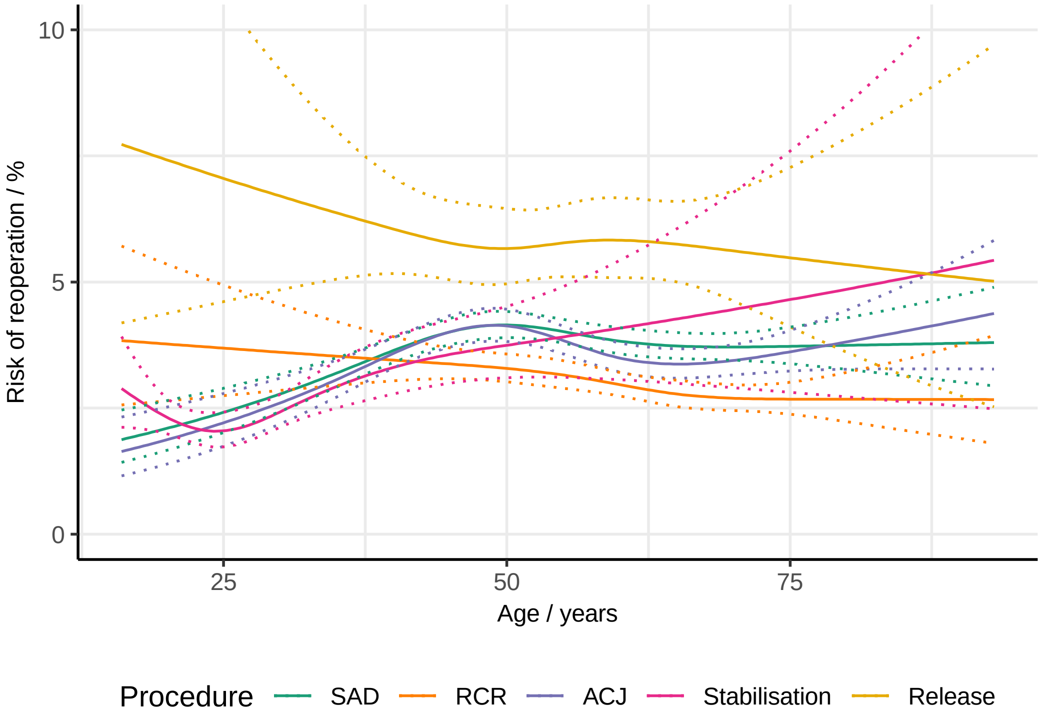

Supplementary figure 3: Partial-effect plot demonstrating the influence of age and procedure type on predicted risk of reoperation within 1 year, adjusted to male sex and Charlson Index of 0.

Supplementary table 2: Coefficients from regression for any reoperation within one year. Values are odds ratios with 95% CIs

Procedure	SAD	RCR	ACJ	Release	Stabilisation
**N**	91928	60463	63262	17810	17955
**Events**	3903	2014	2623	1042	509
**Age contrast**	46 vs 63	53 vs 68	47 vs 64	47 vs 59	22 vs 35
**Age effect**	0.92 (0.84 to 1.01)	0.84 (0.75 to 0.95)	0.82 (0.73 to 0.91)	1.02 (0.87 to 1.21)	1.39 (1.12 to 1.72)
**Male**	Ref	Ref	Ref	Ref	Ref
**Female**	1.15 (1.08 to 1.22)	1.11 (1.02 to 1.22)	1.15 (1.06 to 1.24)	0.92 (0.81 to 1.04)	1.17 (0.94 to 1.44)
**Charlson 0**	Ref	Ref	Ref	Ref	Ref
**Charlson 1**	1.13 (1.05 to 1.23)	1.16 (1.04 to 1.29)	1.15 (1.05 to 1.26)	1.16 (1.00 to 1.34)	1.16 (0.91 to 1.47)
**Charlson 2**	1.06 (0.96 to 1.18)	1.26 (1.11 to 1.44)	1.17 (1.04 to 1.32)	1.13 (0.95 to 1.35)	1.78 (1.12 to 2.83)

Risk of reoperation for infection within 365 days – regression

Outcome: Any reoperation on ipsilateral shoulder for infection within 365 days
Variables: Age, procedure type, sex, diagnosis of diabetes mellitus
Sample size: 251 418
Events: 225
Degrees of freedom in final model: 6

Age was modelled with splines. Knots at 28, 50, 60 and 75 years. Non-linear effects of age were significant and preserved. There was no significant interaction between age and procedure type. Inclusion of ethnic origin and IMD did not significantly alter the coefficients and did not improve the model.

Supplementary table 3: Coefficients from regression for reoperation for infection within 365 days

**Variable**	**Odds ratio (95% CI)**
Age (46 vs 64 years)	2.14 (1.44 to 3.19)
SAD	Reference
RCR	2.75 (1.97 to 3.83)
ACJ	1.09 (0.72 to 1.66)
Stabilisation	1.38 (0.55 to 3.42)
Release	0.34 (0.11 to 1.10)
Male	Reference
Female	0.19 (0.13 to 0.28)
No diabetes	Reference
Diabetes	0.99 (0.68 to 1.34)

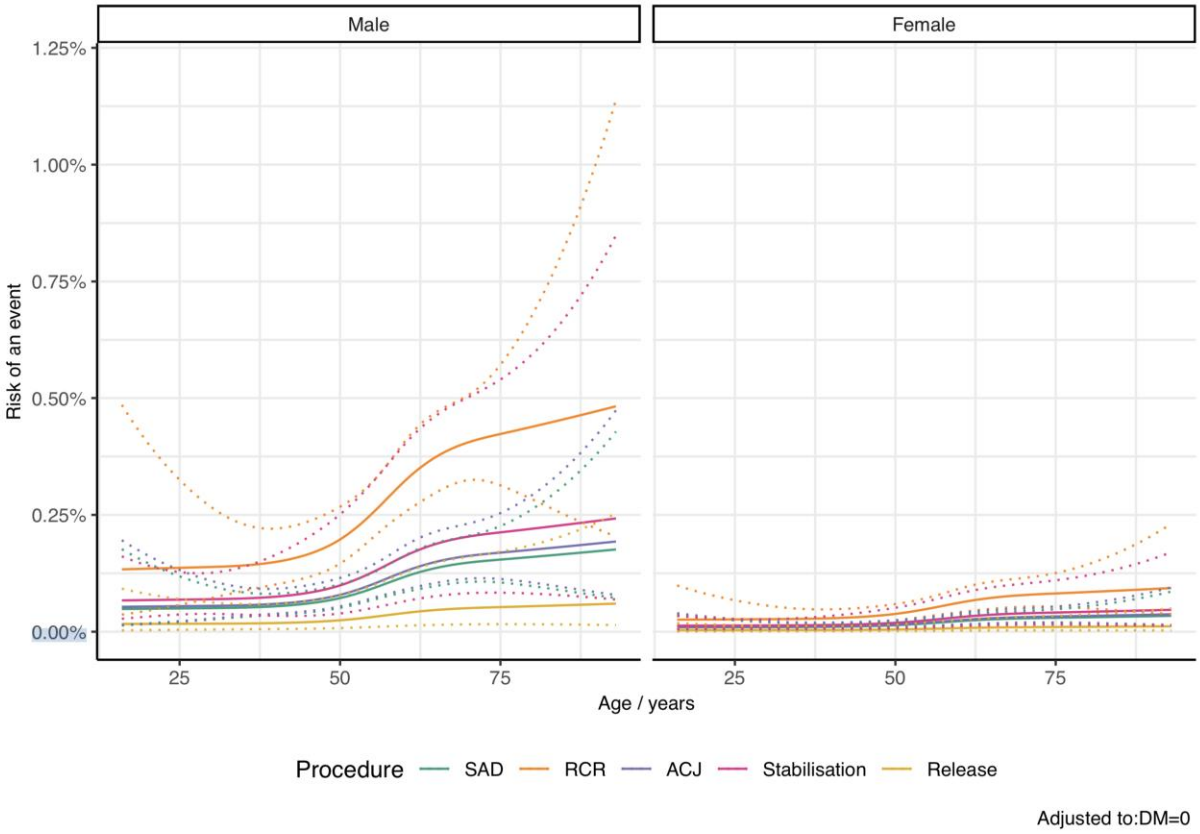

Supplementary figure 4: Partial-effect plot demonstrating the influence of age, sex and procedure type on predicted risk of reoperation for infection within 1 year, adjusted to no diagnosis of diabetes.

Codes used to identify cases and outcomes

OPCS-4 CODES (procedures)

Shoulder girdle anatomic codes (where not otherwise specified)
Z812, Z813, Z814, Z682, Z542

Arthroscopy codes (where not otherwise specified)
W844, W848, W849, W868, W869, Y767, W888, W889, O198, O199

Procedure codes (combined with anatomic codes where necessary)
SAD
O291, W844

RCR
Primary: T791, T794
Revision: T793, T795

ACJ excision
W572, W844, W573

Frozen shoulder release
W781, W784, W788, W789

Stabilisation
O271, O278, O279, O272, O273, O274
W721, W722, W723, W724, W725, W726, W728
W731, W732, W733, W734, W738, W739
W741, W742, W743, W748, W749
W751, W752, W753, W758, W759 (open procedure codes only used for reoperations)
W771, W772, W773, W774, W775, W777, W778, W779
W816, W841, W842

Additional codes used to remove cases and produce a more cleanly defined SAD group
Tenotomy/tenodesis
T70, T645
Chondral procedures
W83, W845, W891, W892, W898, W899, O191
W711, W712, W713, W714
Synovial procedures
T711, W691, W692, W693, W694, W695, W698, W699
Soft tissue recon – SLAP repair
W847

Laterality of surgery codes
Z941, Z942, Z943

Reoperations (in addition to those already stated above)

Debridement/drainage/washout type procedures
T774, T963, W181, W182, W183, W184, W185, W188
W336, W337, W338, W339
W691, W692, W693, W694, W713
W801, W802, W803, W808, W809
W813, W814, W817, W818, W819

Bursal procedures including excision
T621, T622, T626, T628, T629

Excision arthroplasty
W572

Fasciotomy
T551, T552

Arthrodesis
W601, W602, W603, W608, W609
W611, W612, W613, W614, W618, W619
W621, W622, W628, W629
W631, W632, W638, W639
W641, W642, W648, W649

Manipulation or hydrodistension
W911, W913, W918, W919, W922

Others, not specified
W811, W812, W815
W881, W888, W889
W921, W923, W924, W925
O198, O199, W843, W846, W848, W849, W861, W868, W869
W072, W073, W078
W928, W929, W588, W589
O101, W108, W109

Arthroplasty codes (with anatomy codes as needed)
W491, W494, W501, W504, W511, W515, W581
W430, W431, W432, W433, W438, W439
W440, W441, W442, W443, W443, W448, W449
W450, W451, W452, W453, W454, W458, W459
W490, W492, W493, W498, W499
W500, W502, W503, W508, W509
W510, W512, W513, W514, W518, W519
W575, W579
W580, W582
W960, W961, W962, W963, W964, W965, W966, W968, W969
W970, W971, W972, W973, W974, W975, W976, W978, W979

W980, W981, W982, W983, W984, W985, W986, W987, W988, W989
W601, W602, W603, W608, W609
W611, W612, W613, W618, W619
W621, W622, W628, W629
W631, W632, W638, W639
W641, W642, W648, W649
O060, O061, O062, O063, O068, O069
O070, O071, O072, O073, O078, O079
O080, O082, O083, O084, O081, O088, O089
X071, X072
Y037

ICD-10 CODES (diagnoses)

(3 or 4 character where required)

Frozen shoulder (adhesive capsulitis)
M750

Infection
M00, M01, M710, M711, M251, M86, T845, T846, T847, T813, T814

Fractures (shoulder girdle only – for exclusion)
S42

W19, W20, W21, W22, W23, W24, W25, W26, W191, W241, W651, W653, W654, W656, W661, W663, W664, W671, W673, W677, W332, O172, O173, O175, O178, O179 (*OPCS codes for surgical management of fractures)

Dislocation
S430, S431

W652, W658, W689, W662, W668, W669, W672, W674, W678, W679 (*OPCS codes for relocations)

Tumours of interest
C795, C400, M907, M122, C81, C82, C83, C84, C85, C90, D160

Myocardial infarction
I21, I22

Pulmonary embolism
I26

Lower respiratory tract infection
J12, J13, J14, J15, J16, J18, J20, J22, J86, J440, J851, J690

Acute kidney injury
N17

Urinary tract infection
N10, N300, N308, N309, N390

Cerebrovascular event/stroke
I60, I61, I62, I63, I64

Neurovascular injury
T812 (within same spell only)
